# SPARC Fusion Protein Induces Cellular Adhesive Signaling

**DOI:** 10.1371/journal.pone.0053202

**Published:** 2013-01-21

**Authors:** Lamei Cheng, E. Helene Sage, Qi Yan

**Affiliations:** 1 Benaroya Research Institute, Seattle, Washington, United States of America; 2 Department of Biological Structure, School of Medicine, University of Washington, Seattle, Washington, United States of America; 3 Institute of Reproduction and Stem Cell Engineering, Central South University, Changsha, China; UT-Southwestern Med Ctr, United States of America

## Abstract

Secreted protein, acidic and rich in cysteine (SPARC) has been described as a counteradhesive matricellular protein with a diversity of biological functions associated with morphogenesis, remodeling, cellular migration, and proliferation. We have produced mouse SPARC with a FLAG-tag at the N-terminus of SPARC (Flag-SPARC, FSP) in a Bac-to-Bac baculoviral expression system. After affinity purification, this procedure yields SPARC of high purity, with an electrophoretic mobility of ∼44 kDa under reducing conditions, and ∼38–39 kDa under non-reducing conditions. Unexpectedly, FSP adsorbed to plastic supported cell attachment and spreading, in a calcium-dependent manner. The adhesive activity of native FSP was inhibited by prior incubation with anti-SPARC IgG. Cell adhesion to FSP induced the formation of filopodia and lamellipodia but not focal adhesions that were prominent on cells that were attached to fibronectin. In addition, FSP induced the tyrosine phosphorylation of FAK and paxillin in attached epithelial cells. Erk1/2 and Rac were also activated in cells attached to FSP, but at a lower level in comparison to cells on fibronectin. This study provides new insight into the biological functions of SPARC, a matricellular protein with important roles in cell-extracellualr matrix interactions.

## Introduction

SPARC, also known as osteonectin and BM-40, is a matricellular calcium-binding glycoprotein that participates in the regulation of morphogenesis, cell migration/adhesion, and differentiation [1]–[3]. SPARC plays important roles in development, wound healing, bone formation, adipogenesis, angiogenesis, cataractogenesis, and tumor invasion or metastasis [4]–[7]. Mice with a targeted disruption of the SPARC gene exhibit early cataractogenesis, accelerated wound healing, enhanced adipogenesis, and osteopenia [1].

Diverse biological functions have been proposed for SPARC based for the most part on data from experiments in vitro. SPARC has been considered the prototypic counteradhesive matricellular protein, because it induces cell rounding and changes in mesenchymal cell shape that result in the disruption of cell-extracellualr matrix (ECM) interaction. This counteradhesive function of SPARC was defined in vitro with SPARC protein isolated from cultured cells. However, this activity is cell-type dependent, and the source of SPARC protein also appears to be important for its counteradhesive function. For example, SPARC purified from mouse parietal yolk sac (PYS) cells, or recombinant human SPARC (rhSPARC) expressed in *E.coli* elicited rounding of cultured bovine aortic endothelial cells (BAE), fibroblasts, and smooth muscle cells, and inhibited the spreading of newly-plated cells [8]–[10]; however, PYS SPARC did not exhibit the same anti-adhesive effect on F9, PYS-2, and 3T3 cells [1], all of which are transformed lines. In addition, rhSPARC produced by human 293 and HT 1080 cell lines did not show a counteradhesive effect on endothelial cells [11]. Rempel et al. reported that SPARC-transfected glioma cell lines demonstrated increased attachment to collagen and laminin substrates [12]. Another matricellular glycoprotein, thrombospondin (TSP), which is generally considered to be counteradhesive, also exhibits adhesive properties that are dependent on the source of the protein and the target cell type. For example, TSP isolated from human platelets promoted adhesion in vitro of a variety of cells including platelets, melanoma cells, muscle cells, endothelial cells, fibroblasts, and epithelial cells [13]–[14]. TSP synthesized by squamous carcinoma cells also promoted the adhesion of human keratinocytes, fibroblasts, and fibrosarcoma cells [15].

In the present study, we have produced a biologically active FLAG-tagged murine SPARC (FSP) recombinant protein in a baculoviral system. The purity of FSP was greater than 95%. We report here that this FSP enhanced cell attachment and promoted the spreading of lens epithelial cells, bovine aortic endothelial cells (BAE), and murine fibroblasts in vitro. Moreover, FSP promoted the formation of filopodia and lamellipodia and activated proteins of signal-transducing cascades that are involved in focal adhesions. We conclude that SPARC participates in an adhesive signaling pathway in certain cells; this novel activity of SPARC provides new insight into its biological functions as an adhesive protein in cell-extracellular matrix interactions.

## Materials and Methods

### Production and purification of recombinant mouse SPARC with FLAG peptide tag

Mouse (m)SPARC cDNA, minus the signal sequence (amino acids 18–292), was amplified by PCR with mouse lens epithelial cell (mLEC) cDNA as a template: forward primer- 5′-GGGGACAAGTTTGTACAAAAAAGCAGGCTTCGCCCCTCAGCAGACTGAAGTTGCT -3′, and reverse primer- 5′-GGGGACCACTTTGTACAAGAAAGCTGGG TCTTAGATCACCAGATCCTTGTT-3′; attB1 (forward) or attB2 (reverse) overhang sequences were added to create the recombination sites. The amplified fragment was cloned into pDONR™221 vector and subcloned into the expression vector pFBIF derived from pFastBac1 (GATEWAY∧TM Cloning Technology; In Vitrogen). pFBIF contains a fragment encoding the human immunoglobulin signal peptide to allow for efficient secretion of recombinant protein and FLAG peptide. The cloned product was sequenced to confirm proper insertion of the FLAG peptide at the N-terminus of SPARC and the correct sequence of mSPARC. DH10_BAC_ competent cells were transformed with pFBIF containing mSPARC cDNA. Bacmid DNA was isolated from DH10_BAC_ cells and was transfected into *Spodoptera frugiperda* (Sf21) cells to generate recombinant baculovirus. Transfected cell supernate was subsequently used to generate high-titer stocks of recombinant virus for future infections of sf21 cells, which produced conditioned medium containing FSP. The secreted FSP protein was purified on anti-FLAG M1 Agarose Affinity Gel (Sigma, St. Louis, MO) according to the manufacturer's instructions. The integrity of the purified recombinant protein was evaluated by SDS-PAGE under reducing and non-reducing conditions by Coomassie brilliant blue or silver staining, and by Western blot with anti-FLAG M2 antibody (Sigma) or anti-mSPARC antibody (R&D Systems Inc., Minneapolis, MN).

### Cell adhesion assay

Murine lens epithelial cells (mLEC), a mouse lens epithelial cell line established from SPARC-null lens cells [16], immortalized human lens epithelial cells (hLEC) [17], bovine aortic endothelial cells (BAE), and murine lung fibroblasts were grown as described previously [18] and were maintained in Dulbecco's modified Eagle's medium (DMEM) supplemented with 10% fetal bovine serum (FBS), 100 units/ml penicillin G, and 5 uM streptomycin sulfate. Cell attachment assays were performed as described previously [18], [19] with minor modifications. Fibronectin (Fn, Sigma; 10 ug/ml), FSP (5 to 40 ug/ml), rhSPARC (5 to 40 ug/ml) and pure BSA (Pierce, 10 ug/ml) were diluted in DMEM and each was immobilized onto non-tissue culture Titertek-96-well plates by incubation overnight at 4°C. Nonspecific sites were blocked with heat-denatured 1% BSA for 3 hr at room temperature. Trypsinized cells that had been re-suspended in serum-free DMEM were plated on the protein-coated wells at 1×10^5^ cells/well and were incubated for 2 hr at 37°C in a tissue culture incubator. Non-adherent cells were removed by washing twice gently with phosphate-buffered saline containing 1 mM Ca^2+^ and 1 mM Mg^2+^ (PBS^+^). Adherent cells were fixed with 5% glutaraldehyde and stained with 0.1% crystal violet. After extensive washing with deionized water, the bound dye was solubilized with 4% sodium dodecyl sulfate in PBS by the addition of 100 ul to each well. The number of attached cells was quantified by dye extraction and measurement of absorbance at 560 nm in an OPTImax microplate reader (Molecular Devices, Sunnyvale, CA). Each experiment was repeated a minimum of four times, in triplicate. Statistical significance was analyzed by Student's t-test.

### Assay of cell attachment with or without calcium

FSP and Fn were diluted in 10 mM Hepes (pH 7.2), 135 mM NaCl, 3 mM KCl, and 0.5 mM MgCl_2_ (HBS) containing either 1 mM CaCl_2_ or 0.5 mM ethylene glycol tetraacetic acid (EGTA) and were adsorbed onto 96-well plates overnight at 4°C. The wells were washed three times with HBS containing either 1 mM CaCl_2_ or 0.5 mM EGTA. mLEC grown in 10%FBS/DMEM were washed with HBS and incubated briefly in trypsin solution. Trypsin was neutralized with 0.5 mg/ml soybean trypsin inhibitor (Sigma) in HBS. Cells were suspended in HBS with 1 mM CaCl_2_ or 0.5 mM EGTA, plated onto the coated wells at 1×10^5^ cells/well, and allowed to attach at 37°C. Adherent cells were fixed, stained, and photographed as described above.

### Removal of FLAG tag from FSP

To exclude the effect of FLAG peptide on FSP activity, we removed FLAG from the fusion protein with the protease enterokinase (Sigma). FSP/PBS was incubated with 5 units of enterokinase per µg of FSP overnight at 37°C to cleave the FLAG tag according to the manufacturer's instructions.

### Immunofluorescence

Confluent cultures of mLEC that had been serum-starved for 48 hr were released in trypsin solution and resuspended in soybean trypsin inhibitor for 3 hr (Sigma). Cells suspended in serum-free DMEM were plated on coverslips precoated with 10 ug/ml Fn, FSP, or BSA overnight at 4°C and were incubated for 3 hr 37°C. The medium was removed and wells were washed gently with PBS to remove unattached cells. Adherent cells were fixed in 4% paraformaldehyde, made permeable with 0.1% Triton x-100, immunostained with anti- paxillin IgG (1∶2000, BD Bioscience), or anti-FLAG M2 IgG (1∶100, Sigma), followed by secondary antibodies conjugated with Texas-red or FITC (Jackson ImmunoResearch Laboratories, Inc., West Grove, PA). To visualize the actin cytoskeleton, we labeled cells with AlexaFluor 488 phalloidin A (Molecular Probes, Inc.). Immunofluorescence was detected with a Leica microscope equipped for epifluorescence. Images were photographed with a digital camera.

### Immunoprecipitation, immunoblotting, and Rac activation assay

mLEC were grown in 10% FBS/DMEM until subconfluent and were subsequently incubated in serum-free DMEM for 48 hr at 37°C. Cells were released with trypsin (0.125%), incubated with soybean trypsin inhibitor, and re-suspended in serum-free DMEM. Cells were plated on 6-well plates precoated with proteins as described above for 1 hr at 37°C, and were subsequently washed gently with cold PBS. Cells were lysed with Mammalian Protein Extraction Reagent (M-PER; Pierce Biotechnology, Rockford, IL) in the presence of 1 mM sodium orthovanadate (Na_3_VO_4_) and 1 mM sodium fluoride (NaF). The cell lysates were prepared for evaluation of FAK, paxillin, and Erk. For the Rac activity assay, cells were lysed in a Mg^2+^ lysis/wash buffer (25 mM HEPES, pH 7.5, 150 mM NaCl, 1% igepal CA-630, 10 mM MgCl2, 1 mM EDTA, and 2% glycerol) (Upstate Biotechnology) according to the manufacturer's instructions (Upstate Biotechnology). Protein concentrations were determined by a bicinchoninic acid assay (Pierce Biotechnology). Aliquots containing equal amounts of protein were immunoprecipitated with a mouse anti-phosphotyrosine (Tyr) IgG (1 ug/ml; Santa Cruz Biotechnology, Santa Cruz, CA) for FAK and paxillin activity assays, or by the use of a Rac/cdc42 assay kit (PAK-1 PBD, agarose) (Upstate Biotechnology) for Rac-GTP activity analysis. The protein-anti-phosphoTyr IgG immunocomplex was captured by protein A/G agarose beads. Immunoprecipitates were centrifuged and washed three times with Mg^2+^ lysis/wash buffer and subjected to Western blotting.

Immunoprecipitates or cell lysates were separated by SDS-PAGE, transferred to PVDF membranes (Millipore, Billerica, MA), probed with the antibodies described below, and detected by the use of an enhanced chemiluminescence system (Pierce). Focal adhesion-related Tyr-phosphorylated proteins were identified with specific antibodies anti-paxillin IgG and anti-FAK IgG (BD Biosciences). Phosphorylated Erk1/2 and total Erk1/2 were detected with antibodies against phosphor-Erk1/2 and Erk 1/2 (BD Biosciences). Bound Rac1 proteins were detected with anti-Rac1 antibody (Upstate Biotechnology). Total Rac1 was used to normalize the protein in each lane after resolution by SDS-PAGE.

## Results

### Expression and purification of recombinant fusion protein FSP

**Figure 1:**
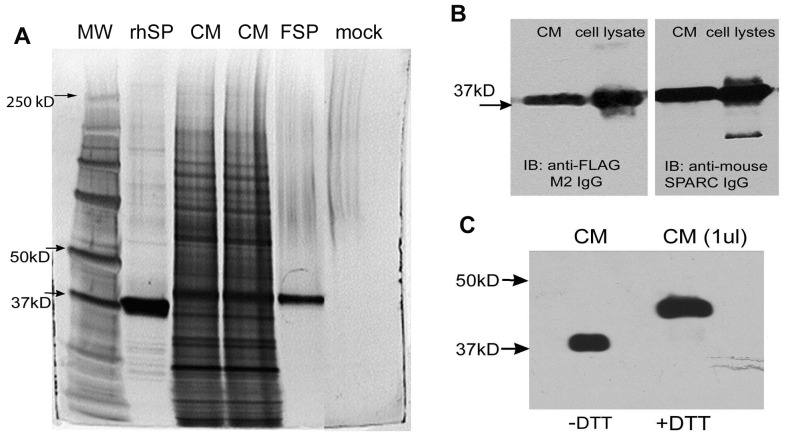
Production and purification of FSP. Conditioned medium (CM) from baculovirus infected Sf21 insect cells was subjected to immuno-affinity chromatography on anti-FLAG M1 affinity gel. CM (15 ul of culture supernate), rhSP (rhSPARC, purified by anion-exchange chromatography), FSP (5 ul of eluted fraction), and mock (buffer control) were separated by SDS-PAGE on a 10% gel under non-reducing conditions, and were visualized by silver stain (**A**); note the other bands in the purified rhSPARC sample, but not in the FSP sample (**B**), 1 ul of CM of infected Sf21 cell lysates (∼5 ug total protein per lane) was resolved by SDS-PAGE under non-reducing conditions, transferred to a PVDF membrane, and probed with anti-FLAG M2 IgG or polyclonal anti-mouse SPARC IgG. (**C**), 1 ul CM was diluted in sample buffer and was boiled for 5 min in the presence (+) or absence (−) of 50 mM DTT. Proteins were resolved by SDS-PAGE, followed by immunoblotting for SPARC. A shift of FSP from Mr ∼38,000 to ∼44,000 was apparent.

The recombinant fusion protein FSP was expressed and secreted into the conditioned medium (CM) of virus-infected Sf21 insect cells. The highest level of FSP expression in the CM was achieved after 4 days of incubation after viral infection. CM was subjected to SDS-PAGE, followed by silver staining. A band was visualized at Mr ∼38,000, among other secreted proteins (Fig. 1A, CM). Immunoblotting with antibodies against mSPARC or FLAG both recognized a band of Mr 38,000 in the CM or cell lysates (Fig. 1B).

Serum-free CM collected from virus-infected Sf21 cells was subjected to affinity chromatography on anti-FLAG M1 agarose. Eluted fractions were separated on SDS-PAGE under non-reducing conditions and were visualized by silver or Coomassie blue staining. FSP was eluted as a single ∼38 kDa band, virtually devoid of any contaminants (Fig. 1A, lane FSP). FSP migrated 1 kD greater than rhSPARC on SDS-PAGE (Fig. 1A). To test that no adhesive proteins such as Fn or laminins were present in the FSP fractions, we performed Western blot analysis with anti-Fn IgG and anti-laminin IgG (Sigma); no signal was seen in the eluted fractions (data not shown). By gel scan, the purity of FSP was greater than 95%. Furthermore, the identity of the purified FSP was confirmed by Western blot with antibodies against SPARC or FLAG. SPARC contains seven disulfide bonds. The apparent Mr of FSP was ∼44,000 under reducing conditions, and ∼38,000 to 39,000 under non-reducing conditions (Fig. 1C), a shift indicative of the intramolecular disulfide bonds in FSP. The approximate yield of purified FSP ranged from 0.6 to 1 mg/10^8^ cells.

### FSP enhanced cell attachment and spreading in vitro

**Figure 2:**
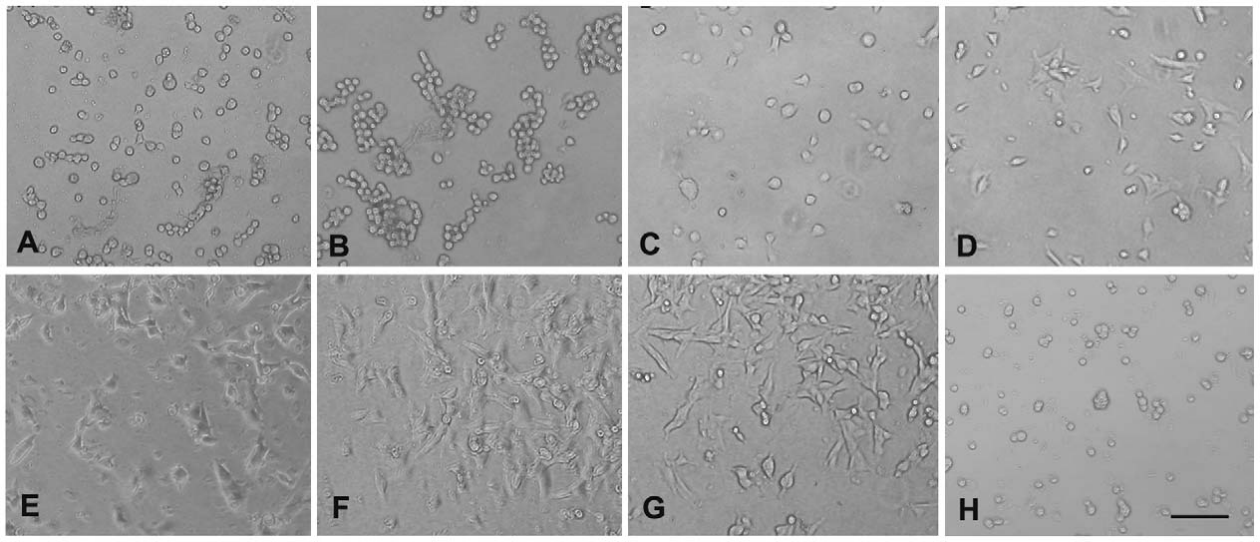
FSP supports mLEC attachment and spreading in a concentration-dependent manner. (A) BSA (10 ul/ml), (B) rhSPARC (10 ug/ml), (C–G) FSP (5, 10, 20, 30, 40 ug/ml, respectively), and (H) FSP (10 ug/ml) were preincubated with anti-SPARC IgG (30 ug/ml) at 37°C for 30 min prior to the coating. The proteins were coated onto wells overnight at 4°C. mLECs were plated into each well in serum-free DMEM, and allowed to attach for 2 hr at 37°C. Phase-contrast photomicrographs were taken on the 96-well plates under an inverted microscope equipped with a digital camera. Scale bar = 50 um for all photographs.

**Figure 3:**
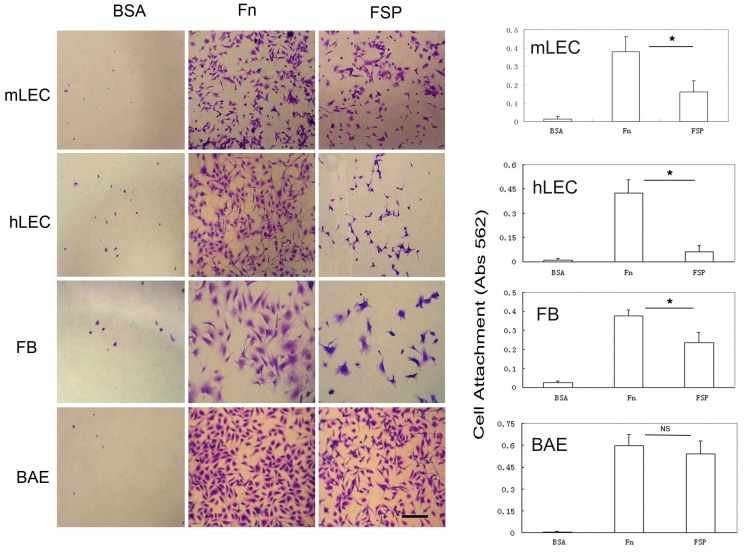
FSP supports cell attachment and spreading. BSA, FSP, and Fn were coated onto wells overnight at 4°C. mLEC, hLEC, murine fibroblasts (FB), and BAE cells were plated into each well in serum-free DMEM, and allowed to attach for 2 hr at 37°C. Cells were stained with 0.1% crystal violet, and representative photographs are shown of cells on each substrate. Cell attachment was also quantified by a colorimetric assay (right panel). The absorbance at 562 nm was correlated directly with the number of cells bound to the substrate. *Bars* represent the mean +/− SD of three experiments carried out in triplicate. *P<0.05; NS, not significant. Scale bar = 50 um for all photographs.

The effect of FSP on cell adhesion/cell spreading was studied in vitro. Human LEC, BAE, mLEC, and fibroblasts were utilized in the assay. Fn is a well-established adhesive protein and was included as a positive control; pure BSA (generally non-adhesive) was included as a negative control. Individual wells of a 96-well plate were coated with BSA (10 ug/ml), Fn (10 ug/ml), and rhSPARC and FSP at different concentrations (5, 10, 20, 30 and 40 ug/ml). mLEC were plated onto each coated well in serum-free DMEM and allowed to attach for 2 hr at 37°C. On BSA-coated wells, there was little, if any, attachment of any of the four cell types tested after 2 hr of incubation (Fig. 2 and 3). Consistently as we previously observed, rhSPARC substrate did not support cell attachment and spreading (Fig. 2B). In marked contrast, FSP (5 to 40 ug/ml) adsorbed onto the wells resulted in a significant increase in cell attachment and spreading in comparison to BSA and rhSPARC substrates after 2 hr of incubation. The number of cells that attached to the wells increased as the concentration of FSP increased from 5 to 20 ug/ml; higher concentrations produced no further change (Fig. 2C–G). The concentration of 10 ug/ml FSP was chosen for the rest of the experiments described below, with four types of cells tested. Cell attachment in wells coated with 10 ug/ml FSP + anti-SPARC antibody (Fig. 2H) was almost identical to that of cells plated onto BSA-coated wells. FSP-mediated adhesive activity was blocked by anti-SPARC antibody. Next, four types of cells were utilized for adhesion assays. Shown in Fig. 3 is an example of wells coated with 10 ug/ml FSP (p<0.001, Fig. 3). Fn promoted a significant increase in the attachment of three cell types (with the exception of BAE cells, *P*>0.05) relative to that of FSP (*P*<0.05, Fig. 3). Over a 2 hr incubation period, the differential extent of cell spreading between Fn and FSP substrates was not as apparent as that of attachment, particularly with respect to BAE cells. Cells incubated on Fn showed better spreading compared to cells on FSP (Fig. 3).

**Figure 4:**
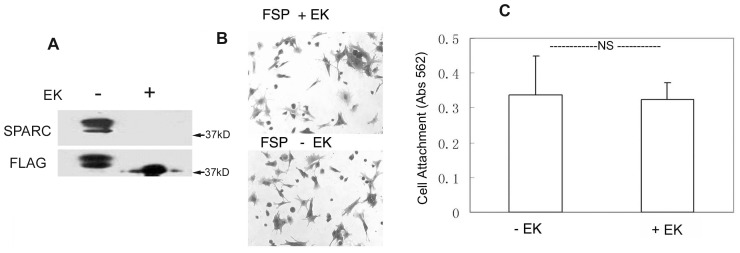
The adhesive effect of FSP was not affected by the FLAG tag. FSP treated with (+) or without (−) enterokinase (EK) was resolved by SDS-PAGE under non-reducing conditions and was immunoblotted with anti-FLAG IgG or anti-SPARC IgG (**A**). FSP (10 ug/ml) with(+) or without (−) EK was coated onto 96-well plates overnight at 4°C. LEC were plated into the wells and incubated in serum-free DMEM for 2 hr at 37°C. Cells were stained with crystal violet (**B**); cell attachment was also quantified by a colorimetric assay at 562 nm (**C**). Results are mean +/− SD of experiments carried out in triplicate. NS, not significant (*P*>0.05).

The unexpected adhesive property of the fusion protein FSP prompted us to ask whether FLAG tag contributed to the adhesive effect of FSP. First, we tested whether this adhesive function was specifically exerted by SPARC by pre-incubating FSP with anti-mouse SPARC antibody (R &D Systems) for 30 min at 37°C prior to coating of the wells. The effect of FSP on the promotion of cell attachment was completely blocked by this anti-SPARC antibody at 20 ug/ml; the non-immune IgG control (20 ug/ml) did not inhibit FSP activity. Coating of SPARC antibody alone had no effect on cell attachment and spreading (data not shown). Secondly, we removed FLAG peptide from the fusion protein FSP with enterokinase (EK), which cleaves the end of the five-amino acid recognition sequence of the C terminus of the FLAG octapeptide. FSP was incubated in the presence or absence of EK overnight at 37°C; removal of the FLAG peptide was confirmed by immunoblotting with anti-FLAG and anti-mSPARC antibodies. A reactive band of a slightly lower molecular weight relative to the EK-untreated, intact FSP, and not recognized by FLAG antibody, was observed (Fig. 4A). In contrast, EK-untreated FSP was revealed by both anti-SPARC IgG and anti-FLAG IgG, with two bands migrating at Mr 38–40,000 (Fig. 4A). It is possible that the incubation overnight at 37°C caused the partial degradation of FSP protein. Furthermore, we repeated the cell adhesion assay described above with FLAG-removed FSP. The FSP without FLAG showed the same effect on mLEC attachment and spreading as that of the original FSP (P>0.05, Fig. 4B & C). Moreover, FLAG peptide itself or FLAG-BAP (Sigma, a FLAG control recombinant protein) showed no effect on cell adhesion under the same culture conditions (data not shown). These results indicate that the adhesive activity of FSP was due to mSPARC rather than to FLAG.

### The effect of calcium on the attachment of cells to FSP

**Figure 5:**
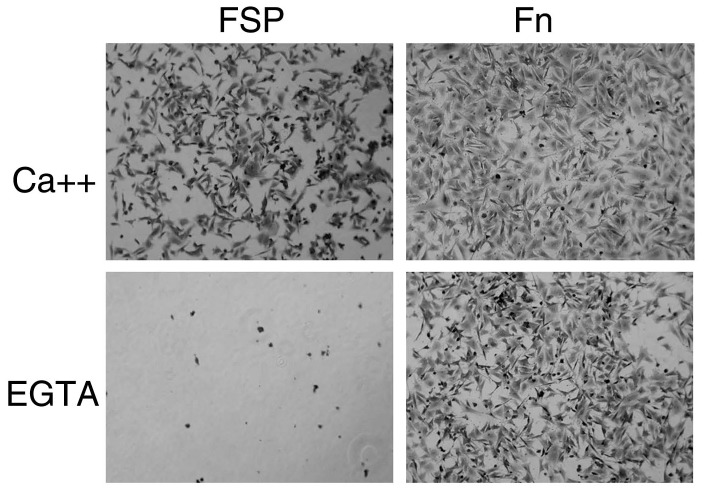
The effect of calcium on the attachment of LEC to FSP. 96-well plates were coated with FSP(10 ug/m) or Fn (10 ug/m) diluted in HBS containing either 1 mM CaCl_2_ or 0.5 mM EGTA for 2 hr at 37°C.Trypsin-released LEC were re-suspended in HBS containing either 1 mM CaCl_2_ or 0.5 mM EGTA and plated on precoated plates for 2 hr at 37°C. Cells were stained with 0.1% crystal violet, and representative photographs are shown.

SPARC is a calcium-binding glycoprotein. Because a major conformational change has been described in SPARC upon binding of calcium ions [20], we investigated the effect of calcium on FSP-mediated cell adhesion. FSP was dissolved in HBS containing either 0.5 mM EGTA or CaCl_2_ and was immobilized onto a 96-well plate. After a 2 hr incubation of mLEC in the coated wells, the cell attachment assay was performed. Cells did not attach to EGTA/FSP coated-wells (Fig. 5). Under the same experimental conditions, the cells were capable of attachment to Fn, albeit at a decreased level of attachment compared to CaCl_2_/Fn (Fig. 5). These results indicated that FSP-mediated cell attachment and spreading were calcium-dependent.

### Cell adhesion to FSP induced activation of FAK/Paxillin, Rac1, and Erk1/Erk2

**Figure 6:**
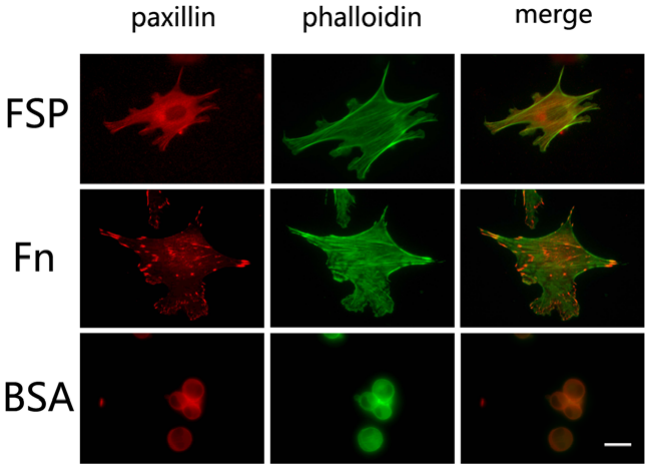
FSP induces the formation of actin stress fibers, filopodia, and lamellipodia. Serum-starved cells were plated on coverslips coated with FSP (**A**), Fn (**B**), or BSA (**C**) for 3 hr at 37°C. Cells were immunostained for paxillin, and were counterstained with phalloidin green. Paxillin was localized in filopodia formed in the cells spreading on the FSP substrate. Scale bar = 8 um for all photographs.

Cell spreading is dependent on the formation of focal adhesions. Focal adhesions were revealed by immunstaining of paxillin, a key focal adhesion-associated adaptor protein. mLEC were plated onto coverslips coated with FSP, Fn, or BSA as described above. After 3 hr incubation in serum-free DMEM, cells that adhered to FSP formed filopodia and lamellipodia, and paxillin was localized to the tips of filopodia (Fig. 6, FSP, white arrows), characteristic of focal plaques. Cells that adhered to Fn exhibited an increased extent of spreading as observed in Fig. 2, and prominent typical focal adhesion structures were distributed over the cells (Fig. 6, Fn). Apparently, cells plated on Fn showed a much stronger adhesion compared to cells on FSP. Cells plated on BSA were rounded and most were detached from the substrate (Fig. 6, BSA). We also added FSP (1∼10 ug/ml) to spreading mLEC cultures - cell shape and the distribution of adhesion plaques were not affected (data not shown).

**Figure 7:**
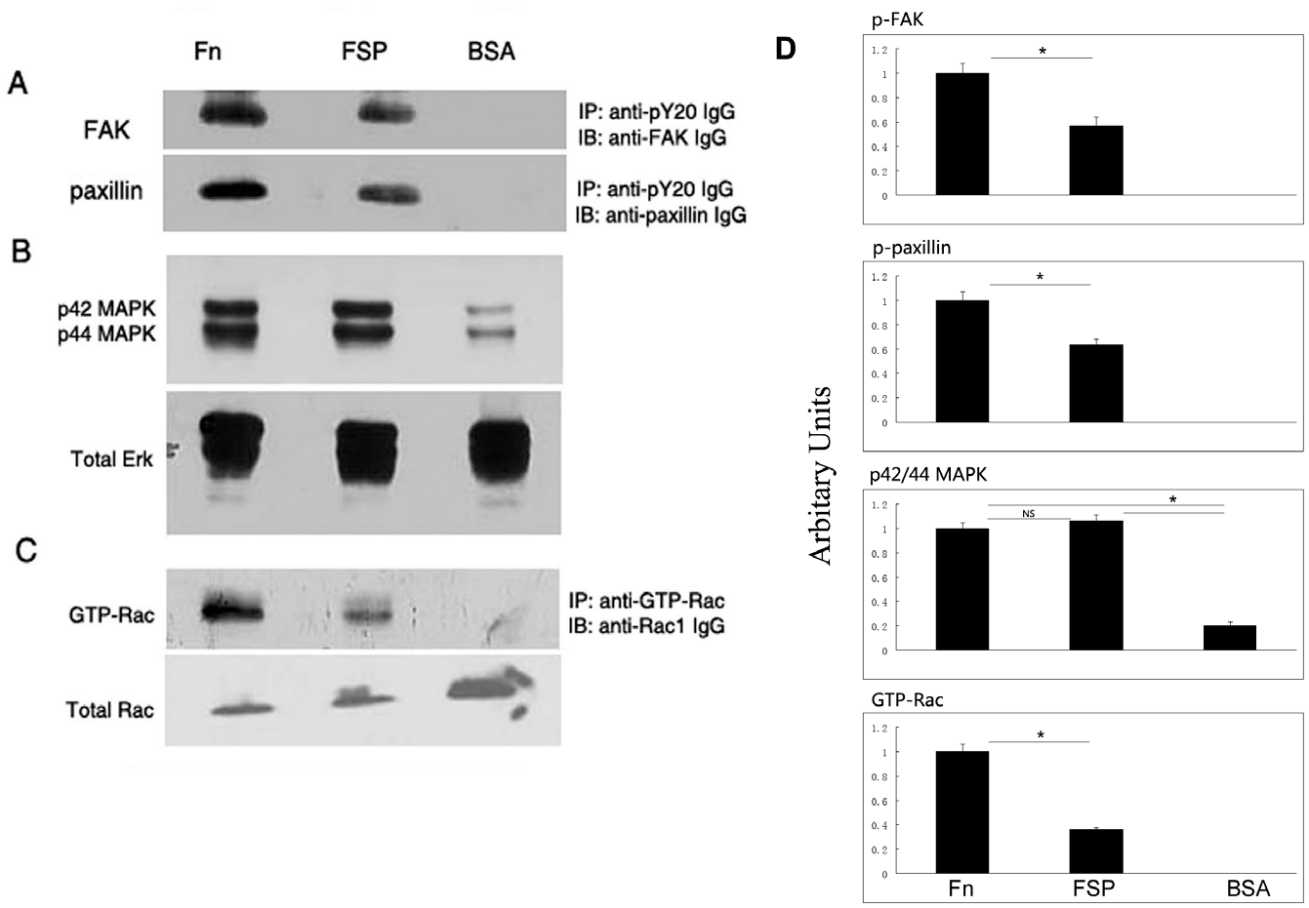
FSP induces phosphorylation of FAK, paxillin, and ERK1/2, and activates the small GTPase Rac in LEC. LEC cultured in serum-free DMEM for 48 hr were plated on dishes precoated with Fn, FSP, or BSA for 1 hr at 37°C. Cell lysates were prepared. (**A**), immunoprecipitation of total cell lysates was performed with an anti-pY20 antibody followed by immunoblotting with anti-FAK IgG or anti-paxillin IgG. (**B**), Total cell lysates were immunoblotted with anti-phospho-Erk-1 (p42 MAPK) and anti- phospho-Erk-2 (p44 MAPK). Subsequently, the membrane was stripped and re-probed with anti-Erk IgG (total Erk). (**C**), Total cell lysates were incubated with anti-GTP-Rac IgG; activated Rac was affinity-precipitated and subsequently immunoblotted with IgG against Rac. An aliquot of cell lysate from each sample was immunoblotted for total Rac protein. (**D**) The histogram on the right shows results of scanning densitometry of p-FAK, p-paxillin, p42/44 MAPK, and GTP-Rac of three experiments with mean +/− SD. Data were normalized to the loading control and were plotted relative to Fn levels. *P<0.05; NS, not significant.

Associated with focal adhesions are numerous signaling proteins including focal adhesion kinase (FAK) and paxillin [21]. FAK is a critical cytoplasmic tyrosine kinase that mediates integrin-mediated signaling following cell adhesion to ECM proteins [22]; paxillin is a substrate of FAK with multiple tyrosine phosphorylation sites, and its activation also plays a key role in the regulation of integrin-mediated signaling [23]. To determine whether substrate-associated FSP could activate phosphorylation of FAK and paxillin, we allowed serum-starved LEC to attach to the FSP-, Fn-, or BSA-coated plates for 1 hr in serum-free DMEM. Cell lysates were immunoprecipitated with anti-phosphotyrosine (py20) antibody and were analyzed by immunoblotting with antibodies recognizing FAK and paxillin. The phosphotyrosine content of FAK and paxillin was undetectable in the serum-starved cells when they were held in suspension in BSA-coated wells (Fig. 7A), but phosphorylation occurred when cells attached to FSP- and Fn-coated wells. Cells plated onto Fn exhibited higher levels of phosphotyrosine for both FAK and paxillin relative to cells plated onto FSP-coated wells (Fig. 7A).

Erk1/Erk2 play a central role in a variety of cell activities such as cell proliferation, differentiation, and adhesion. It has been shown to be activated by integrin-mediated signaling upon cell adhesion to ECM proteins, e.g., Fn [24]. Thus, equal amounts of cell lysates were analyzed by Western blotting with p44/p42 MAPK (Erk1/2) antibodies. Fig. 7B shows that phosphorylated Erk1/Erk2 was elevated significantly in cells attached to FSP; this inductive level was similar to that in cells on Fn (P>0.05). In contrast, cells in BSA-coated wells were mostly round, unattached, and suspended, with their p42/44 significantly decreased (Fig. 7B). The same blots were stripped and re-probed with antibody against the total Erk protein to monitor protein loading. The data indicate that FSP-supported mLEC adhesion resulted in the activation of Erk signaling pathway.

The Rho family GTPase Rac1 is one of the key signaling components controlling actin cytoskeletal organization and formation of lamellipodia subsequent to adhesion to ECM proteins [25]–[26]. To ask whether Rac is activated in cells adhered to FSP, we allowed serum-starved mLEC to attach to precoated dishes in serum-free DMEM for 1 hr; an affinity precipitation with PDB (p21 binding domain from human PAK-1)-glutathione S-transferase fusion protein was used in the precipitation to capture activated Rac from total cell lysates (Upstate Biotechnology). As shown in Fig. 7C, Rac1 was activated in cells plated on Fn and FSP for 1 hr. The level of activated Rac was significantly higher in cells attached to Fn-coated wells in comparison to cells in FSP-coated wells. Cells plated in BSA-coated wells showed undetectable levels of activated Rac.

## Discussion

To obtain highly purified SPARC with biological activity, we expressed mSPARC with FLAG-tag in a Bac-to-Bac™ expression system. It is reported that the FLAG octapeptide allows fusion proteins to retain their original conformation and function and has proven effective for the purification of recombinant proteins [27]. Using anti-FLAG M1 antibody resin, we generated FSP of greater than 95% purity; no other methods had produced SPARC protein with such a high purity (Fig. 1A). FSP migrated on SDS-PAGE at Mr 38,000∼39,000 (Fig. 1A), close to the molecular weight of murine PYS-SPARC (∼40,000). The rhSPARC migrated at Mr 37,000 on SDS-PAGE (Fig. 1A). The difference in Mr between rhSPARC and FSP could have resulted from the different species (mouse versus human), and the post-translational modifications such as glycosylation(s). The acidic amino acid content (pI∼4) also affects the rate of migration and differs between human and mouse SPARC. The process of extraction and purification of FSP was not expected to alter protein structure. It was reported that SPARC produced from the PYS cell line was co-purified with other proteins such as laminin and serum albumin [28]–[29]. rSPARC isolated from *E. coli* can be contaminated by endotoxin, which could cause endothelial cell rounding and apoptosis [30]. Purification of FSP avoided or minimized these problems.

In this study, FSP was found to promote the adhesion of certain cell types such as mLEC, hLEC, BAE cells, and fibroblasts in vitro. FSP immobilized on plastic substrates promoted cell adhesion in a concentration- and calcium- dependent manner, and FSP-mediated adhesive activity was blocked by anti-SPARC antibody (Fig. 2H). This key observation seems inconsistent with our previous reports that SPARC is a counter-adhesive protein. At first, we suspected that FLAG might have contributed to this discordance; however, the experiments in Fig. 4 indicate that FLAG does not alter the adhesive action of FSP. In the past, SPARC produced by different methods had displayed somewhat dissimilar characteristics. For example, SPARC produced by human cell lines did not cause cell rounding or inhibit cell proliferation [9]. This classic counter-adhesive property assigned to SPARC was primarily demonstrated by PYS-SPARC when adding it to attached cells in culture. In this study, rhSPARC and/or FSP were immobilized on plastic. However, each displayed opposite effects in terms of cell attachment and spreading after the cell suspension was added. Although evidence as yet to address this disparate adhesive activity between rhSPARC and FSP is lacking, the following possibilities offer some explanations: 1) The addition of the FLAG peptide to mSPARC might have resulted in subtle conformational differences that affected the biological activity of SPARC; 2) FSP has some different post-translational modifications that could affect some of its biological activities, and 3) FSP has a high degree of purity, devoid of any contaminations that other methods could not avoid; therefore, pure FSP exhibits an adhesive function that previously could have been masked. A recent study showed that SPARC enhances adhesion between somitic myotomes during tailbud development in Xenopus laevis, and directly or indirectly, promotes cell-cell adhesion in vivo [31].

The matricellular proteins have complex actions: some of them both promote and inhibit cell adhesion, and these activities depend on the source of the protein, on protein-protein/receptor interactions, target cells, and the extracellular environment. We have tested blocking antibodies against integrins α_3,_ α_5,_ α_v_β_3,_ and β_1_ on mLEC, but they failed to inhibit FSP-mediated cell adhesion: the FSP-coated wells showed no difference in cell adhesion with or without anti-integrin antibodies (data not shown).

The matricellular protein family consists of TSP, tenascin, osteopontin, SPARC, hevin, and several other secreted proteins. They are structurally diverse but in general promote cell detachment, induce cell rounding, and diminish focal adhesions. However, there are situations in which they perform adhesive rather than counter-adhesive functions. For example, studies have reported that tenascin enhanced endothelial cell attachment and spreading by α_2_β_1_ and α_v_β_3_ integrins [32]; TSP supported the attachment of HUVEC although its adhesive effect was less potent than those of Fn and tenascin substrates [33]; SPARC promotes cell-cell adhesion in Xenopus laevis [31].

Fn is an adhesive glycoprotein and was used as a positive control for this study. Cells spreading on Fn showed strong adhesion, whereas on FSP formed only faint adhesion plaques, filopodia, lamellipodia, and weak stress fibers (Fig. 6). Thus, the adhesive capacity of FSP is much weaker in comparison to the traditional adhesive ECM proteins such as Fn. Recent studies suggested that stabilin-1 and integrin α5 and β3 might mediate some of the effects of SPARC [34]. It is presently unknown whether or which integrin receptors are involved in FSP-mediated cell adhesion. A few integrins (α_3,_ α_5,_ α_v_β_3,_ and β_1_) were tested in our experiments, with negative results. We speculate that cell adhesion to FSP might not be mediated through integrins, as integrin engagement normally produces stronger levels of phospho-FAK, -paxillin, and GTP-Rac; these activities as mediated by FSP were significantly lower in comparison to responses elicited by FN. It could be through modulation of a cell-surface receptor such as demonstrated by SMOC for ligand engagement [35]. Interaction of FSP with a mLEC surface molecule could trigger a cytoplasmic signal resulting in the phosphorylation of FAK and paxillin, and the activation of a series of downstream signaling molecules such as Erk and Rac that leads to cell adhesion and spreading.

SPARC has been described as a multi-faceted protein that modulates cell-cell and cell-ECM interactions. Its divergent actions reveal the complexity of this protein. For example, in cancer, SPARC can act as a tumor suppressor in some tumors, while in others, it is linked with a highly aggressive tumor phenotype [4]. Likewise, depending on the microenvironment, SPARC can act as a de-adhesive or adhesive protein. Our report describing an adhesive property of SPARC provides an explanation for the variable effects of this matricellular protein on cell-ECM interactions. A better understanding of the role of SPARC, for example, in cancer would improve certain biologically-based anti-cancer therapies.
